# Assessment of Bioactive Antioxidants and Anti-Inflammatory Properties of *Apis cerana* L. Honey from Thailand for the Enhancement of Human Health

**DOI:** 10.3390/molecules30183684

**Published:** 2025-09-10

**Authors:** Udomsap Jaitham, Sumed Yadoung, Phannika Tongchai, Peerapong Jeeno, Pichamon Yana, Nid Lungmala, Kanlayanee Boonthawee, Kunrunya Sutan, Khanchai Danmek, Jakkrawut Maitip, Chuleui Jung, Bajaree Chuttong, Surat Hongsibsong

**Affiliations:** 1 School of Health Sciences Research, Research Institute for Health Sciences, Chiang Mai University, Chiang Mai 50200, Thailand; udomsap_j@cmu.ac.th (U.J.); sumed.yadoung@cmu.ac.th (S.Y.); phannika_tongchai@cmu.ac.th (P.T.); peerapong_jeen@cmu.ac.th (P.J.); pichamon.y@cmu.ac.th (P.Y.); nid_lungmala@cmu.ac.th (N.L.); kanlayanee_b@cmu.ac.th (K.B.); 2 Environmental and Occupational Health Sciences Unit, Research Institute for Health Sciences, Chiang Mai University, Chiang Mai 50200, Thailand; kunrunya.s@cmu.ac.th; 3 Office of the University, Chiang Mai University, Chiang Mai 50200, Thailand; 4 School of Agriculture and Natural Resources, University of Phayao, Phayao 56000, Thailand; khanchai.da@up.ac.th; 5 Faculty of Science, Energy and Environment, King Mongkut’s University of Technology North Bangkok, Rayong Campus, Bankhai, Rayong 21120, Thailand; jakkrawut.m@sciee.kmutnb.ac.th; 6 Department of Plant Medicals, Gyeongkuk National University, Andong 36729, Republic of Korea; cjung@andong.ac.kr; 7 Meliponini and Apini Research Laboratory, Department of Entomology and Plant Pathology, Faculty of Agriculture, Chiang Mai University, Chiang Mai 50200, Thailand

**Keywords:** antioxidant activity, phenolic content, flavonoid content, *Apis cerana* honey, functional food

## Abstract

Honey is renowned for its natural antioxidant properties, which help mitigate oxidative stress and lower the risk of diseases such as cardiovascular conditions, cancer, chronic inflammation, and immune dysfunction. This study investigated the antioxidant potential and bioactive compound profiles of 38 *Apis cerana* L. honey samples from Thailand and 2 Manuka honey samples using DPPH, ABTS, and FRAP assays, along with the evaluation of total phenolic and flavonoid contents. The antioxidant activities measured showed a wide range of IC_50_ values, such as the DPPH assay, ranging from 1.59 ± 0.134 mg/L to 824.30 ± 0.64 mg/mL. Manuka honey exhibited the highest antioxidant activity. However, *Apis cerana* L. honey samples, such as sample no. 14, no. 16, and no. 20, showed comparable performance in the ABTS and FRAP. In addition, several samples of *Apis cerana* L. honey, such as no. 12, no. 14, and no. 21, also contain high levels of antioxidants, indicating that *Apis cerana* L. honey has potential as a health food. The results of this study indicate that Thai honey exhibits notable antioxidant capacity and contains significant levels of phenolic and flavonoid compounds, suggesting its potential as a natural dietary source for supporting oxidative stress management. These results indicate that some *Apis cerana* L. honey samples from Thailand have antioxidant properties comparable to Manuka honey. Although differences in floral origin, geographic origin, and bee species should be taken into account, Thai *Apis cerana* L. shows good potential as a natural source of beneficial bioactive compounds. This highlights its potential for use in functional foods and nutritional interventions targeting oxidative stress-related diseases.

## 1. Introduction

Over the past few years, honey has been gaining scholarly interest due to its impressive medicinal values. Besides being a natural sweetener and a health food [1], honey has also been identified as a nutritional source with the capability to regulate a broad spectrum of biological functions [2]. The therapeutic use of bee products, rooted in traditional practices and now supported by scientific evidence, is known as apitherapy. This practice is used to prevent and treat a wide range of diseases, including wounds, arthritis, neurological disorders, immune deficiencies, and gastrointestinal disorders [3,4]. Honey itself possesses a wide range of biological activities, encompassing anti-inflammatory [5], antioxidant [6], antimicrobial, anticancer, antibacterial [7], and antidiabetic [8], among others [6]. Furthermore, its well-documented role in accelerating wound healing and modulating the immune response further underscores its current medical relevance [9].

Fructose and glucose are the primary chemical elements of honey, with trace amounts of phenols, proteins, amino acids, organic acids, and volatile compounds. Phenols are thought to be the main active ingredients in honey [10]. These phenolic chemicals serve as important markers of honey’s quality and place of origin, in addition to adding to its health-promoting qualities [11]. The antioxidant qualities of honey, in addition to its antibacterial activity, have attracted a lot of attention because of their links to anti-inflammatory, anti-cancer, and anti-aging activities [12]. The pharmaceutical, clinical, nutritional, and medical fields have made extensive use of these qualities [13].

The genesis and constituents of naturally occurring honey exhibit considerable variation contingent upon regional and botanical sources [14]. The composition of floral nectar is modulated by a multitude of factors, including geographical location, soil fertility, precipitation, sunlight exposure, elevation, and various other environmental parameters [15]. As a result, the qualitative and biochemical properties of honey are differentiated based on the source of nectar. These discrepancies are frequently ascribed to the specific plant species from which bees harvest nectar, culminating in a range of biochemical profiles characterized by varying concentrations of polyphenolic compounds (including phenolic acids and flavonoids) that exert a substantial influence on the biological activity of honey [16]. Previous research has examined the total phenolic content (TPC) and total flavonoid content (TFC), typically measured in milligrams of gallic acid equivalent (GAE) or milligrams of quercetin equivalent (QE) per 100 g of sample [17]. DPPH radical scavenging activity, ABTS radical scavenging activity, and ferric-reduced antioxidant FRAP are the most commonly used assays to assess antioxidant activity [18]. To gain a deeper understanding of the chemical complexity and antioxidant potential of honey, modern research is also using advanced analysis methods such as LC-MS/MS and HPLC to accurately quantify its bioactive components [19].

In Thailand, a diverse array of honey is generated from various species of honey bees and is widely consumed [20]. Nevertheless, there remains a paucity of research on the biological properties of honey derived specifically from *Apis cerana* L. Previous studies on Apis cerana honey from Southeast Asia show considerable variation in antioxidant and antibacterial potential. For instance, Indonesian honey extracts (Karangasem and Singaraja) demonstrated high phenolic and flavonoid content, strong antioxidant activity (DPPH and FRAP), and antibacterial effects against Bacillus subtilis and Escherichia coli, with positive correlations between phenolic/flavonoid content and bioactivity [21]. Similar regional diversity is reported in Malaysia and Indonesia, where honey types differ by bee species and floral source, yet Apis cerana honey consistently shows health-promoting properties [22]. Despite its international acclaim, the different bioactivity profiles and health benefits of *Apis cerana* L. honey from Thailand have not been sufficiently investigated, leaving a significant gap in the research in this field. The objective of this study was to assess the effectiveness of *Apis cerana* L. honey produced in Thailand. Specifically, the current investigation evaluated the total phenolic content (TPC), total flavonoid content (TFC), and antioxidant properties (utilizing DPPH, ABTS, and FRAP assays) of *Apis cerana* L. honey samples that predominantly originated from the southern region of Thailand. In addition, the volatile profiles of these samples were scrutinized to ascertain unique aromatic compounds that may enhance their sensory attributes and biological efficacy.

## 2. Results

### 2.1. Antioxidant Potential of Apis cerana Honey from Thailand in Comparison to Manuka Honey

Scientific studies acknowledge honey as an antioxidant that helps protect against oxidative stress and diminishes the likelihood of developing chronic illnesses, including cancer and heart disease, as well as immune system problems, cataracts, and inflammation [23]. Spectrophotometric methods such as DPPH, ABTS, and FRAP are typical approaches to evaluate honey’s antioxidant capability, yet no standardized measurement exists for honey’s antioxidant activity. This research conducted comprehensive antioxidant potential analyses on 38 samples of *Apis cerana* L. honey (nos. 1–38) and two samples of Manuka honey (nos. 39 and 40), and the results obtained using these methods are shown in Table 1.

Statistical analysis of antioxidant efficiency demonstrated significant variation among the honey samples, indicating inherent differences in antioxidant capacity across individual sources. Although the botanical origin of the samples was not determined, this variation may be partly attributed to the diverse floral resources naturally available in Pathio District, Chumphon Province, where the samples were collected. The highest antioxidant activity was remarkably demonstrated by Manuka honey (samples 39 and 40), which confirmed its reputation as a premium antioxidant-rich honey with extremely low IC_50_ values for DPPH (1.55–1.58 mg/mL) and high FRAP values (95.75–99.99 mg AAE/100 g). This activity is consistent with its high total phenolic content (47.74–49.40 mg GAE/100 g) and flavonoid content (57.94–62.44 mg QE/100 g). In contrast, several Apis cerana honey samples showed moderate activity (e.g., sample 20 had a DPPH IC_50_ value of 1.59 mg/mL and an FRAP value of 35.01 mg AAE/100 g), indicating that some local honeys can have antioxidant potential comparable to that of Manuka honey. However, some samples, such as 38 and 8, showed relatively weak antioxidant activity, as reflected by their very high IC_50_ values (e.g., sample 38: DPPH = 824.30 mg/mL, ABTS = 172.00 mg/mL).

### 2.2. Total Phenolic Content (TPC) and Total Flavonoid Content (TFC) in Honey Samples

In addition to the antioxidant tests, the total phenolic content (TPC) and total flavonoid content (TFC) of the honey samples were quantitatively assessed. As shown in Table 1, phenolic and flavonoid compounds are recognized as significant contributors to antioxidant efficacy. Phenolic compounds, including flavonoids, phenolic acids, and polyphenols, are powerful antioxidants that help protect against oxidative damage. Honey no. 39 (Manuka honey) had the highest TPC at 49.40 ± 2.66 mg GAE/100 g, which is consistent with the strong antioxidant properties of honey. Furthermore, it is further indicated that Manuka honey contains exceptionally high amounts of these biocomponents, with Manuka honey no. 39 having the highest TFC of 62.44 ± 4.74 mg QE/100 g. The TPC and TFC values of honey no. 39 were significantly higher than the other samples, confirming the classification of this honey as a high-quality honey in terms of antioxidant and health benefits. Among the remaining samples, honey no. 38, which exhibited the highest antioxidant capacity in the spectrophotometric assays, also revealed considerable levels of phenolics at 22.68 ± 2.84 mg GAE/100 g and flavonoids at 13.75 ± 1.11 mg QE/100 g, thereby strengthening the association between these compounds and antioxidant activity. Samples such as Honey nos. 1 and 8 demonstrated lower TPC and TFC values, with phenolic content of 7.51 ± 1.75 mg GAE/100 g and 8.38 ± 5.53 mg GAE/100 g, respectively, suggesting a potentially diminished ability to neutralize free radicals. These findings highlight the importance of TPC and TFC as the main antioxidant components of honey and also highlight that the concentrations of these biocomponents vary significantly between honey types, reflecting their unique origin and production conditions.

### 2.3. Anti-Inflammatory and Hemolytic Activities of Honey Samples

The HRBC membrane stabilization method was used to evaluate the anti-inflammatory and hemolytic activity of 40 honey samples, including Manuka honey (nos. 39 and 40) and several Thai honeys, at a concentration of 100 µg/mL. The anti-inflammatory properties of some Thai honeys were significantly stronger than those of Manuka honey. Many *Apis cerana* L. honey samples may have superior anti-inflammatory qualities compared to Manuka honey at lower concentrations, as shown in Table 2, which shows that honey no. 38 has the highest inhibitory activity at 68.97%, followed by honey no. 37 at 45.10% and honey no. 36 at 44.02%. Honey nos. 39 and 40 had inhibitory percentages of 21.18% and 16.74%, respectively, and had lower percentages of inhibition at the concentration of 500 µg/mL. In general, anti-inflammatory activity was decreased.

However, honey nos. 20, 32, and 35 had inhibitory percentages of 15.08%, 20.29%, and 28.73%, respectively, while honey nos. 39 and 40 had 17.58% and 14.99% and still showed reduced activity. Similarly, at a concentration of 1000 µg/mL, the anti-inflammatory activity was reduced in all samples, but honey nos. 34, 22, and 35 showed 28.04%, 25.47%, and 23.92% and still maintained higher inhibitory activity than honey nos. 39 and 40, which showed 14.43% and 13.69%. In addition to these findings, *Apis cerana* L. honey, especially honey nos. 34, 35, and 36, may be better than Manuka honey for some medical uses because it has bioactive chemicals with anti-inflammatory qualities.

The hemolysis study confirms the possible advantages of Thai honey. When honey no. 38 was diluted to a concentration of 100 µg/mL, honey no. 36 had the lowest hemolysis rate, followed by honey nos. 35 and 34, which showed hemolysis rates of 31.03%, 54.90%, and 55.98%, respectively, indicating that these samples exhibited strong anti-inflammatory activity, with only minor hemolysis. Conversely, Manuka honey nos. 39 and 40 had 78.82% and 83.26% hemolysis, exhibiting significantly higher hemolysis, suggesting that these honeys may induce more hemolysis at the same concentration. The hemolysis rates of each sample increased at concentrations of 500 µg/mL and 1000 µg/mL. However, compared with honey nos. 39 and 40, the Thai honey samples, such as honey no. 34 at 1000 µg/mL, showed lower hemolysis.

## 3. Discussion

### 3.1. Implications of Antioxidant Profiles and Clustering Patterns for Functional and Therapeutic Applications

**Figure 1:**
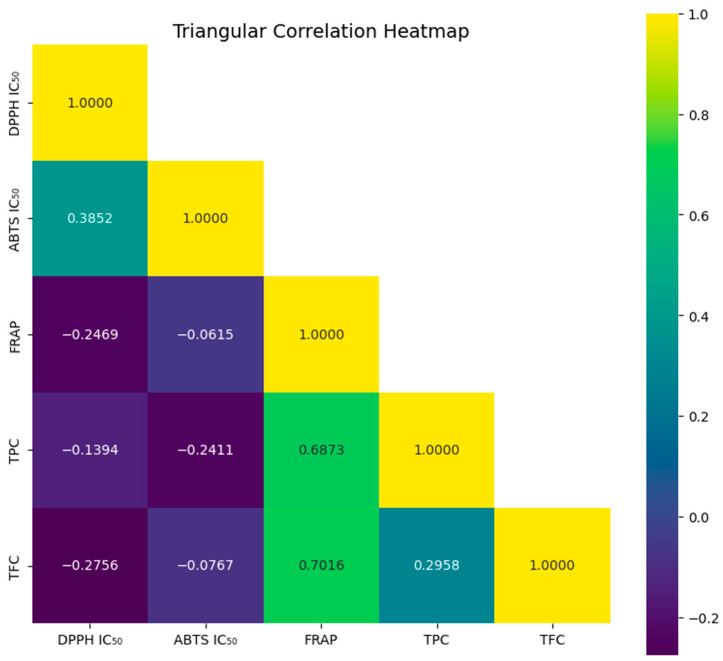
Triangular correlation heatmap of antioxidant activities, total phenolic content (TPC), and total flavonoid content (TFC) in honey bee samples.

This triangular correlation map shows the relationships between antioxidant activity parameters and biological activities in bee samples, specifically DPPH IC_50_, ABTS IC_50_, FRAP, total phenolic content (TPC), and total flavonoid content (TFC). Figure 1 displays negative correlations between the DPPH IC_50_ values and FRAP (−0.2455), TPC (−0.1394), and TFC (−0.2756). This data shows that although lower IC_50_ values indicate higher free radical scavenging efficacy, higher flavonoid and phenolic contents result in lower IC_50_ values, which in turn indicate stronger antioxidant activity. Thus, the role of these biochemicals in antioxidant protection is emphasized by this inverse relationship. This is a biologically significant negative correlation since the lower the DPPH IC_50_ (or the greater the free radical scavenging ability), the higher the FRAP values (or the greater the reducing power). In practice, this implies that honeys rich in phenolic and flavonoids not only exhibit free radical neutralization properties but also provide electrons to stabilize oxidized biomolecules. The same dual activity makes them promising as protective factors against oxidative stress-induced damage to cells, which further supports the usefulness of honey as a natural therapeutic agent. The IC_50_ of DPPH and ABTS have a positive correlation to some extent (0.3852), indicating that although the two tests use different free radical scavenging systems (DPPH and ABTS) to measure antioxidant capacity, the two tests are mechanistically similar. FRAP measures the reducing power of antioxidants, while ABTS measures the free radical scavenging capacity. This data shows that the two tests assess different antioxidant capacities, as indicated by the weaker correlation between FRAP and ABTS IC_50_ (−0.0615).

FRAP test, which assesses the electron donating capacity of antioxidants, showed a strong positive correlation with both TPC (0.6873) and TFC (0.7016), confirming that phenolic and flavonoid compounds play an important role in the reducing energy of bee extracts. These results suggest that bee phenolics and flavonoids are effective electron donors, further supporting the importance of these substances in preventing oxidative stress, since FRAP represents the antioxidant potential to convert Fe^3+^ to Fe^2+^. In addition, TPC and TFC showed a fairly good correlation (0.2958), indicating that bee samples with high flavonoid content were also likely to contain high phenolic compounds. The presence of phenolics and flavonoids together was expected to enhance the total antioxidant potential of bee extracts, as despite their structural differences, they had similar antioxidant processes. After all these factors were considered, this correlation study emphasized the importance of flavonoids and phenolic compounds in the antioxidant phytochemicals of honey.

In addition, TPC and TFC showed a moderate positive correlation (0.2958), indicating that bee samples with high flavonoid content also tend to have high levels of phenolic compounds. Since phenolics and flavonoids have diverse structures but similar mechanisms of action in antioxidant mechanisms, the co-presence of phenolics and flavonoids may enhance the overall antioxidant potential of bee extracts. These studies highlight the importance of phenolics and flavonoids in the antioxidant chemistry of bees. Their potential applications in dietary supplements, functional foods, and pharmaceutical formulations aimed at reducing oxidative damage highlight their contribution to energy reduction, free radical scavenging, and overall protection against oxidative stress.

**Figure 2:**
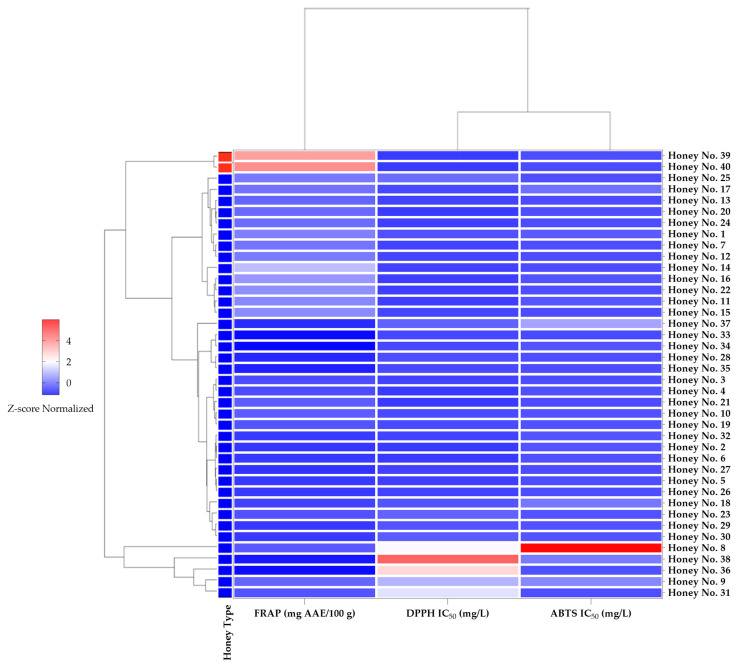
Z-score normalized heatmap and dendrogram of honey samples based on antioxidant properties. The Manuka honey samples (39 and 40) are marked in red, while the Thai honey (*Apis cerana* L.) samples are marked in blue, highlighting the differences between the two honey types.

The honey samples are grouped based on their DPPH IC_50_, ABTS IC_50_, and FRAP values in the hierarchical clustering heatmap displayed in Figure 2. Different groupings were identified by clustering analysis, showing that the antioxidant capacity of the honey samples varied. In this study, spectrophotometric techniques (DPPH, ABTS, and FRAP) were utilized to assess the antioxidant capacity of 38 samples of Thai honey (*Apis cerana* L.) in conjunction with two samples of Manuka honey. The results indicated that Manuka honey exhibited the highest FRAP values, thereby emphasizing its superior reducing capacity. In many cases, these results are consistent with previous studies comparing the antioxidant content of Thai honey (*Apis cerana* L.) with Manuka honey, although several Thai honey varieties showed comparable activities in the DPPH and ABTS tests.

A prior study titled “Comparison of Antioxidant Contents of Thai honey (*Apis cerana* L.) to Manuka Honey” reported that the total phenolic content in Thai honey exhibited substantial variability, ranging from 210 to 1519 mg GAE/kg, with specific varieties such as mangosteen and rambutan honey demonstrating phenolic levels that exceeded those of Manuka honey, which ranged from 563 to 785 mg GAE/kg [24]. Similarly, our findings imply that the composition of bioactive compounds, particularly as indicated by FRAP values, varies markedly among honey samples, with numerous varieties of Thai honey also displaying significant antioxidant potential. Previous research suggests that the floral source and geographical conditions are critical determinants of the antioxidant potential intrinsic to honey [24,25].

In the different antioxidant profiles, the dendrogram structure showed that some Thai honey samples had similar antioxidant properties, while other Thai honey samples showed FRAP values close to Manuka honey. Several Thai honey (*Apis cerana* L.) samples, such as nos. 30, 8, 38, 36, and 9, showed relatively high DPPH IC_50_ values, indicating lower free radical scavenging efficiency. The observed changes in antioxidant activity, as shown by weak or negative correlations with FRAP and positive correlations between DPPH and ABTS values, suggest that the bioactive compounds found in honey work through different pathways. These results are consistent with previous studies that showed that environmental factors, seasonal fluctuations, and nectar content significantly affect the antioxidant properties of honey [26,27].

Antioxidants are essential for scavenging free radicals and reducing oxidative stress, which is linked to long-term conditions like cancer, heart disease, and high blood pressure [7,18]. According to scientific research, honey’s antioxidant qualities help prevent disease by reducing or preventing oxidative damage [28]. Honey is therefore particularly recommended for children, athletes, and immunocompromised individuals, as it promotes overall health and acts as a natural supplement [8].

Our cluster analysis reveals that, despite Manuka honey’s strong antioxidant profile, some Thai honeys also exhibit significant biological activities, suggesting that despite regional and botanical differences, some Thai honey (*Apis cerana* L.) varieties may provide health benefits comparable to those of Manuka honey. These results are in line with previous research conducted in Southeast Asia, where Apis cerana honey from Indonesia (Karangasem and Singaraja) showed potent antibacterial and antioxidant properties. The bioactivities were favorably connected with the amount of flavonoids and phenolic compounds [21]. Similar patterns have been discovered in Malaysia and Indonesia, highlighting the fact that Apis cerana honey continuously demonstrates health-promoting qualities regardless of geographical variances [22].

This heatmap analysis provides clear distinctions between honey types, highlighting the enhanced reducing power of Manuka honey and the variability in Thai honey (*Apis cerana* L.) samples. The changes observed in antioxidant efficacy, as evidenced by weak or negative correlations with FRAP and moderately positive correlations between DPPH and ABTS metrics, suggest that the different biocomponents found in honey act through multiple pathways. These findings are consistent with previous research showing that the antioxidant properties of honey are significantly influenced by the nectar source, seasonal variations, and environmental factors [27,29]. Consequently, when incorporated into dietary regimens, the variety of antioxidant mechanisms found in honey may provide broad protection against oxidative stress, enhancing honey’s importance as a functional health food [28].

The observed variability in antioxidant activity, expressed through moderate positive correlations between DPPH and ABTS values, together with negative or weak correlations with FRAP, suggests that the diverse bioactive compounds present in honey act through multiple mechanisms. These observations corroborate previous studies showing that nectar source, seasonal variation, and environmental determinants strongly influence the antioxidant properties of honey [20,30]. Thus, the multifaceted antioxidant mechanisms present in honey may provide important protection against oxidative stress when incorporated into food products, thus increasing the importance of honey as a health-promoting agent [31].

### 3.2. Total Phenolic Content (TPC) and Total Flavonoid Content (TFC)

**Figure 3:**
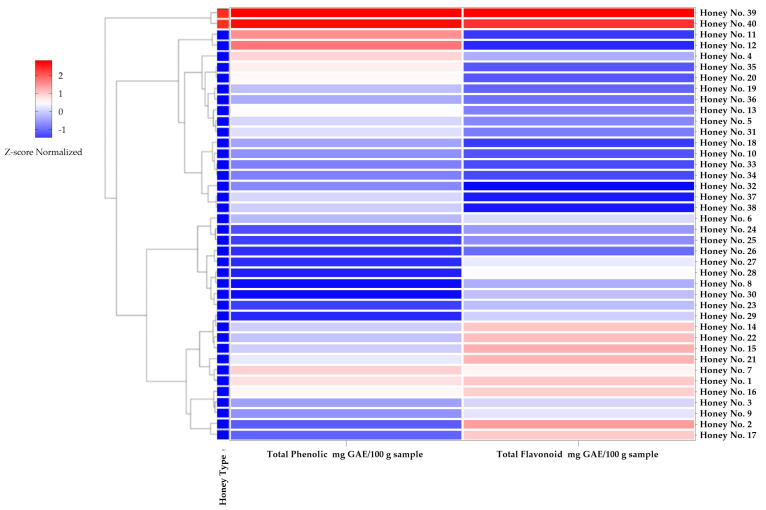
Zscore normalized heatmap and dendrogram of honey samples based on TPC and TFC.

The Z-score-adjusted heatmap in Figure 3 more clearly illustrates the distribution of TPC and TFC in different honey samples, highlighting groups of honey with similar biocomposition. This analysis revealed considerable variability in the composition of bioactive compounds, highlighting the influence of botanical and geographical origin. This clustering indicates that some Thai honey (*Apis cerana* L.) samples have phenolic and flavonoid profiles similar to those of Manuka honey, which may affect the antioxidant potential of the honey.

In the analyzed samples, honey nos. 39 and 40 (Manuka honey) exhibited the highest TPC levels, with 49.40 ± 2.66 and 47.74 ± 3.90 μg GAE/g, respectively, and TFC of 62.44 ± 4.74 and 57.94 ± 9.52 μg QE/g, respectively. In contrast, the Thai honey (*Apis cerana* L.) samples showed a wide range of values; some samples, such as no. 30 TPC honey, had GAE values of 7.48 ± 0.38 μg and TFC values of 29.73 ± 4.08 μg QE/g, and no. 8 TPC honey had GAE values of 7.51 ± 1.75 μg and TFC values of 28.38 ± 5.53 μg QE/g, indicating relatively low TPC and TFC values; the hierarchical clustering analysis clearly separated the Manuka honey samples from Thai honey (*Apis cerana* L.).

However, even with significantly higher TPC and TFC, some Thai honey (*Apis cerana* L.) varieties showed competitive bioactive compound contents even with TFC values lower than 20 μg QE/g. Notably, the TPC values of Thai honey nos. 11 and 12 were relatively high, with values of 35.62 ± 4.06 and 38.96 ± 12.44 μg GAE/g, respectively. Similarly, honey nos. 14, 15, and 22 exhibited TFC levels higher than 40 μg QE/g, which is comparable to the values found in Manuka honey. These results challenge the notion that Manuka honey is superior in bioactive properties and suggest that certain Thai honey varieties possess substantial TPC and TFC, indicating their potential as natural sources of antioxidants with health benefits similar to those of Manuka honey. Previous research analyzing honey samples from Malaysia, Turkey, and Yemen also reported variations in TPC and TFC values, with pine honey and acacia honey displaying the highest values (*p* < 0.05) [32]. Compared with these results, the Thai honey samples in this study showed higher variability, with some samples having TPC and TFC values higher than the reported levels of Malaysian honey.

Moniruzzaman et al. found that the TPC values for Tualang and Acacia honey were 0.35 ± 0.81 μg GAE/mg and 0.19 ± 0.84 μg GAE/mg, while the TFC values were 0.07 ± 0.74 and 0.02 ± 1.73 μg catechin equivalent/mg honey, respectively [33]. The observed differences in the composition of honey highlight the important influence of floral and geographical origin, as well as environmental factors, on the concentrations of phenolic and flavonoid constituents [34,35]. These biocomponents play an important role in the antioxidant capacity of different types of honey, with polyphenols and flavonoids being associated with the inhibition of alpha-amylase activity, as suggested by Devarajan and Venugopal [36]. Furthermore, Kanjiro and Kanjiro [37] reported that flavonoids, such as luteolin, myricetin, and quercetin, found in honey, have the ability to inhibit alpha-amylase activity, suggesting that polyphenol extracts of honey may slow down the hydrolysis of starch to glucose, which may help reduce the risk of diabetes and obesity.

### 3.3. Anti-Inflammatory and Hemolytic Activities of Honey

**Figure 4:**
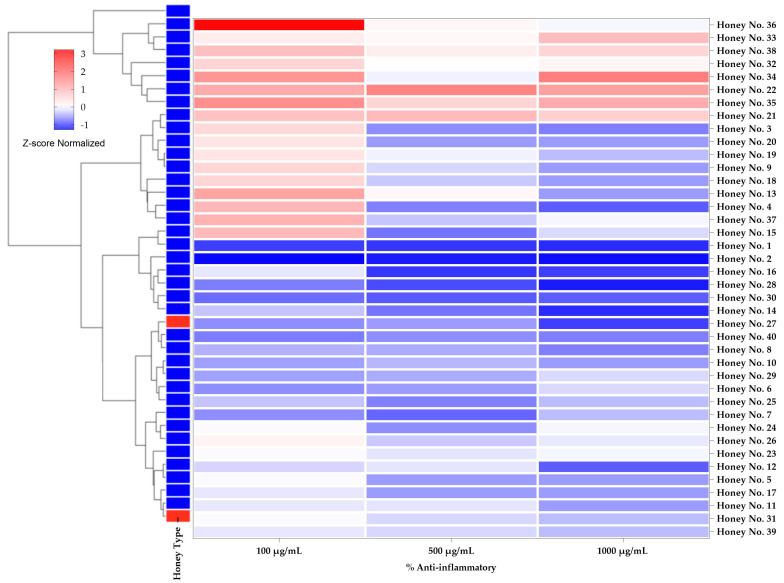
Heatmap representing the anti-inflammatory activity (Z-score normalized) of Thai and Manuka honey samples at different concentrations (100, 500, and 1000 µg/mL). Darker red shades indicate higher anti-inflammatory activity.

**Figure 5:**
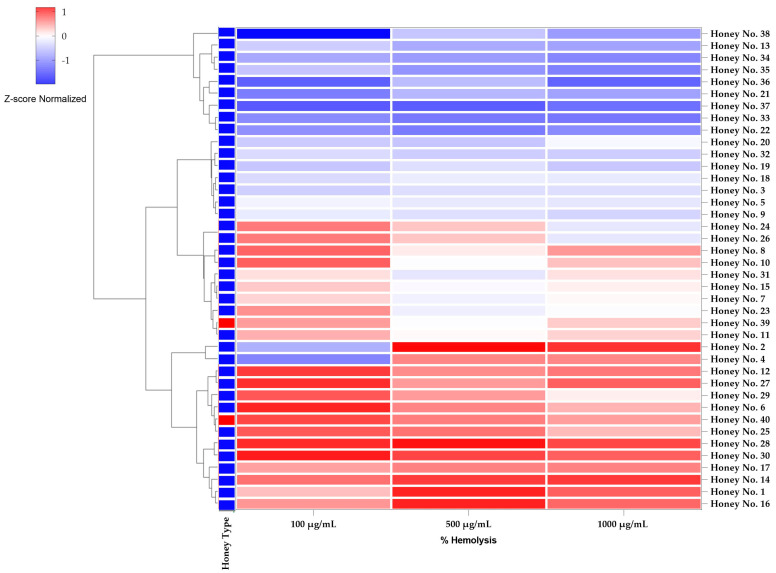
Heatmap representing the hemolysis activity (Z-score normalized) of Thai and Manuka honey samples at different concentrations (100, 500, and 1000 µg/mL). Darker blue shades indicate lower hemolysis, while red shades indicate higher hemolysis.

Normalized Z-score heatmaps with hierarchical clustering dendrograms were used to show the differences in anti-hemolytic and anti-inflammatory activities in Figure 4 and Figure 5 by subtracting the mean and dividing by the standard deviation. Normalizing the Z-scores normalizes the data, allowing direct comparisons of honey samples at different concentrations, where positive Z-scores (in red) indicate high activity and negative Z-scores (in blue) indicate low activity. This technique draws attention to patterns in the dataset. The clustering dendrogram reveals a link between anti-erythropoietic and anti-inflammatory properties, grouping honey samples with comparable biological responses. In particular, honeys with high anti-inflammatory activity tended to cluster together, while honeys with low anti-erythropoietic activity clustered together, highlighting the importance of considering safety for therapeutic use.

Thai honey exhibited anti-inflammatory and hemolytic activities comparable to those of Manuka honey (nos. 39 and 40). The heatmap in Figure 4 shows the anti-inflammatory effects of honey at different concentrations (100, 500 and 1000 μg/mL). In particular, honey nos. 36, 35, and 34 exhibited the highest anti-inflammatory activity at a concentration of 100 μg/mL, with inhibition rates of 68.9%, 45.1%, and 44.0%, respectively. These results indicate that some Thai honeys have a high potential to reduce inflammation, which may be useful in the treatment of inflammatory diseases such as arthritis, wound healing, and chronic inflammation [38]. The bioactive compounds in honey, particularly polyphenols and flavonoids, have been reported to modulate the inflammatory pathway by inhibiting pro-inflammatory cytokines and oxidative stress [39].

The anti-inflammatory characteristics revealed in the current study correspond with earlier research that illustrates how natural honey, abundant in flavonoids, can effectively inhibit inflammatory mediators, including TNF-α and IL-1β, while concurrently downregulating the activity of inducible nitric oxide synthase (iNOS) and the production of reactive oxygen species (ROS) [40]. Additionally, investigations concerning Eucalyptus globulus essential oil and honey have demonstrated that these natural substances can affect the cyclooxygenase and lipoxygenase pathways, exhibiting mechanisms akin to those of synthetic anti-inflammatory agents, albeit without significant adverse effects [41]. Ruiz-Ruiz et al. documented that the extract derived from Melipona bee honey can inhibit albumin denaturation and assist in membrane stabilization, thereby suggesting potential anti-inflammatory properties [42].

Furthermore, Biluca et al. performed an in vitro study utilizing lipopolysaccharide-stimulated RAW264.7 macrophages and discovered that honey can enhance the secretion of the anti-inflammatory cytokine IL-10, thereby indicating an anti-inflammatory effect [43]. These findings are congruent with our results, which demonstrate that certain samples of Thai honey exhibit substantial anti-inflammatory activity, thereby implying the therapeutic viability of Thai honey as an alternative to synthetic pharmaceuticals.

At a dose of 100 μg/mL, Honey nos. 36, 35, and 34 demonstrated greater levels of hemolysis (31.0%, 54.9%, and 55.9%, respectively), despite their strong anti-inflammatory properties (Figure 5). Concerns about the cytotoxic consequences of these honey samples at higher concentrations were seriously raised by the severe hemolysis. This finding holds significant value since honey that exhibits exceptional anti-inflammatory properties and increased hemolytic activity could need to undergo additional purification or dosage optimization to guarantee safe therapeutic usage. Depending on its dosage and biocomposition, natural honey can have cytotoxic effects, as previous research has shown. Manuka honey, for instance, contains higher concentrations of methylglyoxal (MGO), which may be cytotoxic but has also been linked to antibacterial and anti-inflammatory qualities [42].

On the other hand, honey no. 22 exhibited moderate anti-inflammatory effects (33.4% inhibition) while maintaining a low hemolysis rate (66.5% at 100 μg/mL), suggesting that it might be a safer option for therapeutic applications. Similar to honey nos. 13, 4, and 2, various Thai honeys have demonstrated encouraging anti-inflammatory qualities while preserving a comparatively low hemolysis rate, showing that honey no. 2 may be used by humans with few adverse effects [43]. Manuka honey (honey nos. 39 and 40) showed therapeutic properties, showing stable levels of hemolysis (78.8–86.3%) and slight anti-inflammatory activity (16.7–21.1%) at all doses tested. These results are consistent with previous research indicating that the bioactive constituents of Manuka honey, particularly MGO, contribute to its anti-inflammatory effect, although Manuka honey does not have the highest anti-inflammatory activity. Nonetheless, Manuka honey has become generally regarded as a natural medicine due to its low cytotoxicity and well-known wound-healing qualities [44].

In addition, several of the examined honey samples had stronger anti-inflammatory activity than Manuka honey. This study raises the possibility that Thai honey has natural anti-inflammatory qualities. On the other hand, careful safety assessment is required [45], especially considering potential hemolysis and toxicity, to minimize side effects. For example, honeys such as honey nos. 36, 35, and 34, which have strong anti-inflammatory properties but improved hemolysis, may need to be purified or reformulated. Furthermore, honeys with a good safety profile, such as honey nos. 22 and 13, may be more suitable for use as a treatment, as they have only minor anti-inflammatory activity and less hemolysis.

## 4. Materials and Methods

### 4.1. Materials

The chemicals and reagents used in this study were sourced from various suppliers. 2,2-Diphenyl-1-picrylhydrazyl (DPPH) and 2,2′-Azino-bis(3-ethylbenzothiazoline-6-sulfonic acid) (ABTS) were purchased from Sigma-Aldrich (St. Louis, MO, USA). Methanol (HPLC grade, ≥99%) and ethanol (HPLC grade, ≥99%) were obtained from Sigma-Aldrich. Potassium persulfate (K_2_S_2_O_8_) and ferric chloride hexahydrate (FeCl_3_·6H_2_O) were purchased from LOBA CHEMIE PVT., Ltd. (Mumbai, India). Ascorbic acid (purity ≥ 99%) was obtained from Sigma-Aldrich, while gallic acid (GAE) (purity ≥ 98%) was purchased from Sigma-Aldrich. Sodium carbonate (Na_2_CO_3_), sodium nitrite (NaNO_2_), aluminum chloride (AlCl_3_), and sodium hydroxide (NaOH) (1 M) were acquired from ACI Labscan (Bangkok, Thailand). Folin–Ciocalteu reagent was obtained from Sigma-Aldrich, and Quercetin (purity ≥ 98%) was sourced from Sigma-Aldrich. The standards used in this analysis included Trolox (purity ≥ 98%) from Sigma-Aldrich, and Quercetin was used to determine total flavonoid content (TFC). Reagents for buffer preparation included acetic acid and sodium acetate for the FRAP assay.

The following equipment was used for the assays: a spectrophotometer for absorbance measurements; a microplate reader (Tecan, Männedorf, Switzerland) with wavelengths of 510 nm, 515 nm, 593 nm, 734 nm, and 760 nm; 96-well microplates (Corning Inc., Corning, NY, USA), flat-bottomed, polystyrene plates; adjustable micropipettes (10 µL–1000 µL) and sterile pipette tips for liquid handling; volumetric flasks (10 mL, 100 mL), beakers, and graduated cylinders for accurate solution preparation; a magnetic stirrer with a heating plate; an analytical balance (precision ±0.0001 g); a vortex mixer for sample mixing; an incubator for dark incubation steps; a pH meter for buffer preparation; and an ultrasonic bath for reagent mixing when necessary.

### 4.2. Sample Collection

Thirty-eight honey samples from *Apis cerana* L. bees were fitted from local beekeepers in Pathio District, Chumphon Province, Thailand, in January and February 2023. From among these sources, several samples were chosen from beekeeping plots managed and supported by RAKPAA Social Enterprise, a community-based organization promoting sustainable apiculture in the region. To serve as standards for comparison, we purchased two samples of commercial Manuka honey from a certified supplier (Comvita, Te Puke, New Zealand) and included them in the experiment. The honey samples were all shipped to the laboratory while being carefully controlled to keep them in good shape. The samples were kept at 4 °C after arrival and before more analysis was carried out.

### 4.3. Antioxidant Analysis

The antioxidant capability of 40 samples of fresh Thai honey was determined. All samples (2 g) were dissolved in 100 mL of distilled water, followed by heating at 80 °C in order to be completely dissolved and homogenized. The solutions were allowed to cool to room temperature and then filtered using Whatman No. 1 filter paper in order to eliminate particulate matter. The antioxidant properties, such as free radical scavenging capacity, were then evaluated and compared between processed and raw honey samples using conventional spectrophotometric methods.

#### 4.3.1. DPPH Assay for Radical Scavenging Activity

The antioxidant activity of the honey samples was determined using the DPPH (2,2-diphenyl-1-picrylhydrazyl) assay, following the method described and modified by Jeeno et al. [46], with modifications, using ascorbic acid as a standard. A stock solution of 500 µg/mL was prepared by dissolving 5 mg of ascorbic acid in 10 mL of distilled water, and serial dilutions ranging from 0 to 100 µg/mL were made for calibration. The DPPH stock solution was prepared by dissolving 24 mg of DPPH in 100 mL of 100% methanol. The working solution was obtained by diluting 10 mL of the stock solution in 45 mL of methanol to achieve an absorbance of 1.1 at 515 nm. The honey samples were diluted using the twofold dilution method until eight wells were plated in order. Then, 100 µL of each sample or ascorbic acid standard was pipetted into a 96-well plate, with distilled water serving as the blank. Following this, 100 µL of working DPPH solution was added to each well. The plates were incubated in the dark for 30 min at room temperature, and the absorbance was measured at 515 nm. The percentage of inhibition was calculated using the following Formula (1):

DPPH % inhibition = [(A_control − A_sample)/A_control] × 100(1)

where A_control is the absorbance of the control (blank) and A_sample is the absorbance of the sample.

The IC_50_ value, which represents the concentration at which 50% inhibition occurs, was determined based on these measurements.

#### 4.3.2. ABTS Assay for Radical Scavenging Activity

ABTS radical scavenging activity was measured using a modified method based on Arnao et al. [47]. In brief, the analysis was conducted with Trolox as the standard. To prepare the ABTS solution, 19.5 mg of ABTS and 3.3 mg of K_2_S_2_O_3_ were dissolved in 7 mL of distilled water and incubated in the dark at room temperature for 12 to 16 h. A stock solution of Trolox (5000 µM) was made by dissolving 12.9 mg of Trolox in 1.5 mL of 100% ethanol and diluting it with distilled water to a final volume of 10 mL. Working standards with concentrations ranging from 1000 µM to 0 µM were prepared. The ABTS solution was then diluted with 80% ethanol to a 1% concentration, and its absorbance was adjusted to 0.7 ± 0.02 at 734 nm. To determine the radical scavenging activity, 30 µL of the sample or Trolox standard was added to a 96-well plate, followed by 170 µL of the prepared ABTS solution. The plate was incubated in the dark for 10 min, and absorbance was measured at 734 nm. The percentage inhibition was calculated using the Formula (2):

ABTS % inhibition = [(A_control − A_sample)/A_control] × 100(2)

where A_control is the absorbance of the control and A_sample is the absorbance of the sample.

The IC_50_ value, which represents the concentration at which 50% inhibition occurs, was determined based on these measurements.

#### 4.3.3. FRAP Assay for Radical Scavenging Activity

The Ferric Reducing Antioxidant Power (FRAP) assay, adapted from Ben Ahmed et al. [48], briefly evaluates antioxidant activity through redox reactions, observing the color change from ferric tripyridyltriazine (Fe^3+^-TPTZ) to ferrous tripyridyltriazine (Fe^2+^-TPTZ) as antioxidants donate electrons. The results are expressed as FRAP values in mM Fe^2+^/g extract, calculated using a ferrous sulfate standard curve. The assay involves preparing reagents such as acetate buffer (pH 3.6), 10 mM TPTZ in 40 mM HCl, and 20 mM FeCl_3_·6H_2_O, combined in a 10:1:1 ratio to create a clear light brown FRAP reagent. Ascorbic acid standards (0–1000 µg/mL) are prepared. For analysis, 10 µL of standard or sample was added to a 96-well plate, mixed with 190 µL of FRAP reagent, and incubated in the dark for 30 min at room temperature. Absorbance was then measured at 593 nm, and antioxidant capacity was calculated using the formula

FRAP (mg AAE/100 g) = CAA × V × DF × 100/Sample weight (g)(3)

where CAA is the ascorbic acid concentration derived from the standard curve, V is the volume of the extract solution, and DF is the dilution factor.

#### 4.3.4. Total Phenolic Content

The total phenolic content (TPC) was determined using the Folin–Ciocalteu method, adapted from the protocols established by Folin, Ciocalteu, and Dewanto [49]. Gallic acid was used as the reference standard, and the results were expressed as milligrams of gallic acid equivalent (GAE) per gram of dry sample weight. For the analysis, 12.5 µL of the sample was mixed with 13 µL of deionized (DI) water. To this, 13 µL of Folin–Ciocalteu reagent was added, and the mixture was incubated for 6 min. After incubation, 125 µL of a 7% sodium carbonate solution and 100 µL of distilled water were added to the mixture. The absorbance of the solution was measured at 760 nm using a spectrophotometer microplate reader. The total phenolic content was quantified in milligrams of gallic acid equivalents (GAE) per 100 g of the sample. The TPC was calculated using the following formula

TPC (mg GAE/100 g) = CGA × V × DF × 100/Weight of crude extract (g)(4)

where CGA is the concentration of gallic acid from the standard curve (mg/mL), V is the volume of extract solution (mL), and DF is the dilution factor.

#### 4.3.5. Total Flavonoid Content

The total flavonoid content (TFC) was determined using a colorimetric assay based on the method described by [50]. Briefly, 25 µL of the sample solution was added to a 96-well plate, followed by the addition of 8 µL of a 7% sodium nitrite solution and 13 µL of distilled water. The resulting solution was left to stand at room temperature for 5 min. After this incubation, 15 µL of 10% aluminum chloride solution was added to the mixture, which was then thoroughly mixed and incubated at room temperature for an additional 5 min. Next, 50 µL of 1 M sodium hydroxide solution and 28 µL of distilled water were introduced into the mixture, and the solution was incubated at room temperature for 5 min. The absorbance was measured at 510 nm using a spectrophotometer, with distilled water serving as the blank reference. The TFC was calculated and expressed as milligrams of quercetin equivalents per 100 g of sample (mg QE/100 g) using the following formula

TFC (mg QE/100 g) = CQE × V × DF × 100/Weight of crude extract (g)(5)

where CQE is the concentration of quercetin from the standard curve (mg/mL), V is the volume of the extract solution (mL), and DF is the dilution factor.

### 4.4. Anti-Inflammatory Assay (HRBC Membrane Stabilization Method)

The anti-inflammatory assay (HRBC membrane stabilization method) was adapted from Chippada et al. [51]. The honey samples were prepared at concentrations of 1000, 500, and 100 µg/mL. To prepare red blood cells (RBCs), Alsever’s solution was added to the blood collected from volunteers, followed by gentle mixing. Red blood cells (RBCs) were obtained from leftover anonymized samples from a previous research project approved by the Human Experimentation Committee (HEC), Faculty of Associated Medical Technology, Chiang Mai University, in accordance with the Declaration of Helsinki (Certificate No. AMSEC-66EX-062 8/60, 3 November 2023). No new blood collection was performed for this study, and no identifiable personal information was available to the researchers. The NaCl solution was washed through the RBCs and centrifuged at 3000 rpm at 4 °C. The RBCs were washed and then resuspended in NaCl to make a 10 percent *v*/*v* suspension. In the assay, 2 mL of NaCl solution, 0.5 mL of the RBC suspension, and 0.5 mL of honey sample were placed in test tubes, and 1 mL of phosphate buffer (pH 7.4) was added. Tubes were incubated at 37 °C for 30 min and centrifuged at 3000 rpm for 10 min. The absorbance of the supernatant at 560 nm was determined. Controls contained RBC suspension with distilled water instead of honey. Membrane stabilization and hemolysis percentages were calculated using standard formulas. The percentages of membrane protection and hemolysis were calculated using the following equations:

Membrane Protection (%) = 100 − [(A_1_/A_0_) × 100] (6)

Hemolysis (%) = (A_1_/A_0_) × 100 (7)

where A_0_ is the absorbance of the control and A_1_ is the absorbance of the sample.

### 4.5. Statistical Analysis

Each experiment was performed in triplicate, and the results were expressed as mean ± standard deviation (SD). Data were normalized using Z-score transformation to enable comparison across different antioxidant and bioactivity assays. Statistical analyses were carried out using IBM SPSS Statistics version 29.0.1.0 (IBM Corp., Armonk, NY, USA). Statistical significance was determined based on *p* < 0.05.

## 5. Conclusions

In conclusion, this study presents a detailed investigation of the antioxidant and anti-inflammatory properties of Thai honey (*Apis cerana* L.) in comparison with Manuka honey, which is important for human health applications. The results show that some Thai honeys also have remarkable bioactivities, making them suitable alternatives to Manuka honey, despite Manuka honey having higher total phenolic and flavonoid content and better antioxidant capacity. Antioxidant assessment using the DPPH, ABTS, and FRAP methods revealed different profiles in the honey samples, with Thai honey reflecting variability dependent on floral and geographical factors. In addition, the anti-inflammatory properties of some Thai honey samples were comparable to or better than those of Manuka honey, indicating their potential for the treatment of inflammation.

In addition, the ability of Thai honey to inhibit key inflammatory biomarkers in vitro confirms its potential anti-inflammatory activity. Previous studies have reported that flavonoids and phenolic acids, which are present in honey, are recognized for their anti-inflammatory properties, suggesting that honey, including Thai honey, may have health-promoting effects related to inflammation. The health-promoting properties of Thai honey are further emphasized by the association between antioxidant and anti-inflammatory activities. All results suggest that traditional Thai honey can be used as a functional food with medical benefits, but further studies are needed to confirm these health benefits in human populations. Future research should focus on extracting specific biocomponents and conducting clinical trials.

## Figures and Tables

**Table 1:** Results of antioxidant activity analysis and bioactive compound profiling.

Honey Sample	Antioxidant Activities			Bioactive Compound	
	DPPH	ABTS	FRAP	Total Phenolic	Total Flavonoid
	IC_50_ (mg/mL)	IC_50_ (mg/mL)	mg AAE/100 g	mg GAE/100 g Sample	mg QE/100 g Sample
No. 1	41.10 ± 1.66	55.5 ± 1.64	40.4 ± 2.61	29.47 ± 3.60	41.74 ± 4.59
No. 2	2.24 ± 1.37	34.61 ± 0.81	23.95 ± 1.82	13.89 ± 1.60	46.54 ± 4.24
No. 3	12.78 ± 0.62	7.60 ± 2.90	28.6 ± 1.28	19.15 ± 1.78	31.79 ± 3.10
No. 4	1.99 ± 0.33	20.37 ± 1.60	28.5 ± 1.72	30.45 ± 2.70	28.26 ± 0.83
No. 5	4.82 ± 1.03	8.33 ± 2.63	24.35 ± 1.54	23.43 ±1.87	24.63 ± 4.41
No. 6	2.46 ± 1.13	18.72 ± 1.80	24.43 ± 4.66	21.06 ± 2.87	32.59 ± 6.46
No. 7	15.87 ± 0.48	13.93 ± 3.97	34.73 ± 3.31	30.80 ± 3.78	36.98 ± 2.68
No. 8	396.51 ± 0.82	1734.16 ± 1.33	31.25 ± 11.24	7.51 ± 1.75	28.38 ± 5.53
No. 9	257.10 ± 0.75	214.27 ± 1.05	34.15 ± 8.32	18.16 ± 1.69	33.11 ± 0.69
No. 10	22.70 ± 0.62	23.49 ± 1.02	31.70 ± 6.22	18.06 ± 3.88	19.69 ± 2.76
No. 11	5.55 ± 1.96	47.56 ± 0.71	41.79 ± 4.32	35.62 ± 4.06	17.09 ± 2.64
No. 12	17.34 ± 0.89	7.66 ± 0.15	39.35 ± 8.41	38.96 ± 12.44	15.55 ± 1.32
No. 13	11.62 ± 0.88	6.11 ± 0.46	34.12 ± 5.94	26.35 ± 1.32	23.98 ± 1.00
No. 14	10.23 ± 0.95	5.95 ± 0.46	54.38 ± 7.27	22.73 ± 1.43	42.06 ± 1.69
No. 15	17.12 ± 1.52	5.77 ± 0.53	42.60 ± 3.74	22.61 ± 3.33	44.54 ± 1.84
No. 16	4.75 ± 0.62	24.12 ± 1.41	45.66 ± 3.05	27.38 ± 1.12	41.09 ± 3.56
No. 17	22.12 ± 3.67	134.34 ± 0.72	37.21 ± 6.50	14.49 ± 1.65	41.69 ± 4.48
No. 18	49.3 ± 0.35	154.96 ± 1.82	26.19 ± 2.74	19.62 ± 3.17	17.46 ± 0.69
No. 19	30.4 ± 0.75	19.86 ± 1.24	30.42 ± 3.07	21.65 ± 3.28	21.23 ± 1.92
No. 20	1.59 ± 0.134	11.33 ± 0.76	35.01 ± 13.61	27.09 ± 1.18	19.98 ± 2.51
No. 21	1.82 ± 0.79	9.91 ± 0.50	32.17 ± 2.51	24.78 ± 3.45	43.93 ± 2.01
No. 22	3.41 ± 1.40	11.13 ± 1.82	44.02 ± 4.39	21.83 ± 0.94	42.99 ± 3.16
No. 23	81.85 ± 1.27	25.95 ± 2.21	29.49 ± 3.35	11.55 ± 0.91	29.44 ± 4.77
No. 24	5.23 ± 3.28	8.07 ± 2.45	36.29 ± 7.66	12.80 ± 0.86	26.14 ± 0.53
No. 25	106.94 ± 1.74	13.00 ± 0.69	38.61 ± 11.77	11.61 ± 0.97	25.16 ± 0.60
No. 26	12.13 ± 0.54	12.88 ± 0.40	24.43 ± 2.72	10.42 ± 1.60	21.83 ± 2.63
No. 27	10.18 ± 0.67	5.74 ± 1.34	22.70 ± 1.27	10.10 ± 0.45	33.63 ± 0.7
No. 28	33.14 ± 0.56	14.58 ± 0.58	20.15 ± 8.87	9.12 ± 0.56	35.39 ± 1.86
No. 29	46.35 ± 1.27	22.58 ± 5.23	24.14 ± 12.11	9.73 ± 0.46	31.16 ± 3.33
No. 30	65.02 ± 1.98	36.53 ± 0.76	24.37 ± 2.66	7.48 ± 0.38	29.73 ± 4.08
No. 31	345.03 ± 2.09	12.19 ± 1.36	30.66 ± 3.10	24.07 ± 1.34	23.51 ± 3.23
No. 32	12.22 ± 0.73	37.32 ± 1.20	24.63 ± 5.35	17.35 ± 1.11	12.40 ± 0.78
No. 33	20.52 ± 0.91	3.22 ± 2.30	13.61 ± 0.44	16.65 ± 1.00	18.78 ± 4.17
No. 34	21.37 ± 1.31	30.08 ± 0.30	13.00 ± 0.56	16.67 ± 1.63	18.44 ± 1.51
No. 35	27.09 ± 1.63	8.94 ± 2.87	18.43 ± 2.57	28.03 ± 1.43	19.98 ± 1.51
No. 36	505.49 ± 0.54	20.36 ± 0.47	14.19 ± 1.10	20.08 ± 1.64	22.19 ± 4.41
No. 37	78.17 ± 0.42	321.82 ±0.99	21.23 ± 0.77	23.47 ± 4.57	14.13 ± 0.35
No. 38	824.30 ± 0.64	172.00 ± 0.80	16.79 ± 1.24	22.68 ± 2.84	13.75 ± 1.11
No. 39	1.58 ± 1.58	10.32 ± 0.74	95.75 ± 4.12	49.40 ± 2.66	62.44 ± 4.74
No. 40	1.55 ± 2.97	6.82 ± 1.66	99.99 ± 4.88	47.74 ± 3.90	57.94 ± 9.52

**Table 2:** In vitro anti-inflammatory activity analysis of honey using the human red blood cell (HRBC) membrane stabilization method.

Honey Sample	Conc. Honey (µg/mL)	% Hemolysis	% Anti-Inflammatory
		Mean ± SD	Mean ± SD
No. 1	100	77.18 ± 8.20	22.82 ± 8.20
	500	86.89 ± 0.42	13.11 ± 0.42
	1000	87.44 ± 0.57	12.56 ± 0.57
No. 2	100	67.37 ± 4.57	32.63 ± 4.57
	500	87.35 ± 0.27	12.65 ± 0.27
	1000	88.27 ± 0.16	11.73 ± 0.16
No. 3	100	71.22 ± 12.51	28.78 ± 12.51
	500	83.87 ± 1.14	16.13 ± 1.14
	1000	86.98 ± 0.42	13.02 ± 0.42
No. 4	100	63.52 ± 8.91	36.48 ± 8.91
	500	84.88 ± 0.48	15.12 ± 0.48
	1000	86.71 ± 0.32	13.29 ± 0.32
No. 5	100	75.71 ± 5.90	24.29 ± 5.90
	500	84.14 ± 1.14	15.86 ± 1.14
	1000	85.79 ± 1.30	14.21 ± 1.30
No. 6	100	84.88 ± 0.48	15.12 ± 0.48
	500	84.88 ± 0.48	15.12 ± 0.48
	1000	85.98 ± 0.55	14.02 ± 0.55
No. 7	100	75.07 ± 10.88	24.93 ± 10.88
	500	83.23 ± 5.72	16.77 ± 5.72
	1000	85.79 ± 1.11	14.21 ± 1.11
No. 8	100	81.76 ± 0.32	18.24 ± 0.32
	500	82.95 ± 1.20	17.05 ± 1.20
	1000	86.43 ± 0.42	13.57 ± 0.42
No. 9	100	71.13 ± 5.77	28.87 ± 5.77
	500	82.77 ± 4.37	17.23 ± 4.37
	1000	85.70 ± 0.48	14.30 ± 0.48
No. 10	100	81.94 ± 1.04	18.06 ± 1.04
	500	82.49 ± 2.94	17.51 ± 2.94
	1000	85.70 ± 0.95	14.30 ± 0.95
No. 11	100	79.19 ± 5.80	20.81 ± 5.80
	500	84.33 ± 0.99	15.67 ± 0.99
	1000	85.79 ± 0.32	14.21 ± 0.32
No. 12	100	83.85 ± 3.47	16.15 ± 3.47
	500	84.74 ± 1.54	15.26 ± 1.54
	1000	87.05 ± 1.08	12.95 ± 1.08
No. 13	100	61.40 ± 1.06	38.60 ± 1.06
	500	79.33 ± 2.02	20.67 ± 2.02
	1000	86.60 ± 0.67	13.40 ± 0.67
No. 14	100	81.10 ± 6.05	18.90 ± 6.05
	500	86.34 ± 2.92	13.66 ± 2.92
	1000	88.11 ± 0.77	11.89 ± 0.77
No. 15	100	76.22 ± 5.03	23.78 ± 5.03
	500	85.09 ± 2.77	14.91 ± 2.77
	1000	85.36 ± 0.70	14.64 ± 0.70
No. 16	100	79.15 ± 4.00	20.85 ± 4.00
	500	86.87 ± 1.37	13.13 ± 1.37
	1000	87.31 ± 1.26	12.69 ± 1.26
No. 17	100	78.62 ± 4.69	21.38 ± 4.69
	500	84.92 ± 2.92	15.08 ± 2.92
	1000	86.87 ± 1.31	13.13 ± 1.31
No. 18	100	71.61 ± 0.41	28.39 ± 0.41
	500	81.81 ± 3.25	18.19 ± 3.25
	1000	86.96 ± 1.62	13.04 ± 1.62
No. 19	100	72.76 ± 9.82	27.24 ± 9.82
	500	80.66 ± 5.44	19.34 ± 5.44
	1000	86.34 ± 1.20	13.66 ± 1.20
No. 20	100	72.76 ± 7.48	27.24 ± 7.48
	500	84.92 ± 1.81	15.08 ± 1.81
	1000	86.96 ± 0.80	13.04 ± 0.80
No. 21	100	75.60 ± 6.41	24.40 ± 6.41
	500	76.49 ± 4.98	23.51 ± 4.98
	1000	81.01 ± 5.99	18.99 ± 5.99
No. 22	100	66.55 ± 2.93	33.45 ± 2.93
	500	66.99 ± 0.96	33.01 ± 0.96
	1000	74.53 ± 4.30	25.47 ± 4.30
No. 23	100	79.46 ± 2.54	20.54 ± 2.54
	500	81.96 ± 0.48	18.04 ± 0.48
	1000	84.55 ± 0.42	15.45 ± 0.42
No. 24	100	80.57 ± 0.73	19.43 ± 0.73
	500	83.63 ± 0.28	16.37 ± 0.28
	1000	83.90 ± 0.56	16.10 ± 0.56
No. 25	100	82.24 ± 0.48	17.76 ± 0.48
	500	85.20 ± 0.58	14.80 ± 0.58
	1000	85.85 ± 0.28	14.15 ± 0.28
No. 26	100	75.39 ± 7.12	24.61 ± 7.12
	500	83.35 ± 1.94	16.65 ± 1.94
	1000	84.37 ± 1.60	15.63 ± 1.60
No. 27	100	84.37 ± 0.85	15.63 ± 0.85
	500	84.46 ± 0.56	15.54 ± 0.56
	1000	87.42 ± 1.12	12.58 ± 1.12
No. 28	100	84.64 ± 1.05	15.36 ± 1.05
	500	87.14 ± 0.42	12.86 ± 0.42
	1000	87.88 ± 1.05	12.12 ± 1.05
No. 29	100	82.33 ± 1.12	17.67 ± 1.12
	500	84.46 ± 0.73	15.54 ± 0.73
	1000	84.92 ± 0.58	15.08 ± 0.58
No. 30	100	85.48 ± 0.70	14.52 ± 0.70
	500	86.12 ± 1.27	13.88 ± 1.27
	1000	87.42 ± 1.05	12.58 ± 1.05
No. 31	100	80.57 ± 5.85	19.43 ± 5.85
	500	82.79 ± 2.90	17.21 ± 2.90
	1000	85.20 ± 0.58	14.80 ± 0.58
No. 32	100	71.08 ± 8.25	28.92 ± 8.25
	500	79.71 ± 5.15	20.29 ± 5.15
	1000	83.43 ± 0.90	16.57 ± 0.90
No. 33	100	74.41 ± 3.11	25.59 ± 3.11
	500	79.31 ± 1.96	20.69 ± 1.96
	1000	78.92 ± 2.40	21.08 ± 2.40
No. 34	100	55.98 ± 6.12	44.02 ± 6.12
	500	80.59 ± 2.33	19.41 ± 2.33
	1000	71.96 ± 8.69	28.04 ± 8.69
No. 35	100	54.90 ± 11.62	45.10 ± 11.62
	500	71.27 ± 5.97	28.73 ± 5.97
	1000	76.08 ± 2.64	23.92 ± 2.64
No. 36	100	31.03 ± 26.83	68.97 ± 26.83
	500	79.31 ± 3.31	20.69 ± 3.31
	1000	82.16 ± 1.11	17.84 ± 1.11
No. 37	100	62.75 ± 8.78	37.25 ± 8.78
	500	81.27 ± 1.45	18.73 ± 1.45
	1000	82.16 ± 1.89	17.84 ± 1.89
No. 38	100	65.39 ± 15.24	34.61 ± 15.24
	500	77.16 ± 1.72	22.84 ± 1.72
	1000	80.88 ± 0.59	19.12 ± 0.59
No. 39	100	78.82 ± 2.58	21.18 ± 2.58
	500	82.42 ± 1.53	17.58 ± 1.53
	1000	85.57 ± 0.73	14.43 ± 0.73
No. 40	100	83.26 ± 0.85	16.74 ± 0.85
	500	85.01 ± 0.00	14.99 ± 0.00
	1000	86.31 ± 0.42	13.69 ± 0.42

## Data Availability

The original contributions presented in the study are included in the article; further inquiries can be directed to the corresponding authors.
